# Detection of Stroke with Retinal Microvascular Density and Self-Supervised Learning Using OCT-A and Fundus Imaging

**DOI:** 10.3390/jcm11247408

**Published:** 2022-12-14

**Authors:** Samiksha Pachade, Ivan Coronado, Rania Abdelkhaleq, Juntao Yan, Sergio Salazar-Marioni, Amanda Jagolino, Charles Green, Mozhdeh Bahrainian, Roomasa Channa, Sunil A. Sheth, Luca Giancardo

**Affiliations:** 1 Center for Precision Health, School of Biomedical Informatics, University of Texas Health Science Center at Houston (UTHealth), Houston, TX 77030, USA; 2 Department of Neurology, UTHealth McGovern Medical School, UTHealth, Houston, TX 77030, USA; 3 Institute for Stroke and Cerebrovascular Diseases, UTHealth, Houston, TX 77030, USA; 4 Center for Clinical Research and Evidence-Based Medicine, UTHealth McGovern Medical School, UTHealth, Houston, TX 77030, USA; 5 Department of Ophthalmology and Visual Sciences, School of Medicine and Public Health, University of Wisconsin-Madison, Madison, WI 53705, USA

**Keywords:** stroke, optical coherence tomography angiography, deep learning

## Abstract

Acute cerebral stroke is a leading cause of disability and death, which could be reduced with a prompt diagnosis during patient transportation to the hospital. A portable retina imaging system could enable this by measuring vascular information and blood perfusion in the retina and, due to the homology between retinal and cerebral vessels, infer if a cerebral stroke is underway. However, the feasibility of this strategy, the imaging features, and retina imaging modalities to do this are not clear. In this work, we show initial evidence of the feasibility of this approach by training machine learning models using feature engineering and self-supervised learning retina features extracted from OCT-A and fundus images to classify controls and acute stroke patients. Models based on macular microvasculature density features achieved an area under the receiver operating characteristic curve (AUC) of 0.87–0.88. Self-supervised deep learning models were able to generate features resulting in AUCs ranging from 0.66 to 0.81. While further work is needed for the final proof for a diagnostic system, these results indicate that microvasculature density features from OCT-A images have the potential to be used to diagnose acute cerebral stroke from the retina.

## 1. Introduction

In the United States alone, more than 795,000 people suffer from acute stroke annually. It is one of the leading causes of death and disability in the industrialized world. Out of all strokes, 87% are ischemic (blood flow to the brain is obstructed), and the other 13% involve hemorrhagic events (blood vessels rupture in the brain) [1]. While treatments for acute stroke exist, their efficacy is directly connected to the speed with which they can be delivered after stroke onset, with diminishing effect over time. This limitation poses significant challenges, as patients need to be rushed to stroke centers in order to have their brain imaged with a computed tomography (CT) or magnetic resonance imaging (MRI) scanner to confirm the stroke diagnosis and identify any hemorrhage.

Due to the homology between retinal and cerebral vessels and the ease with which retinal images can be acquired non-invasively, retinal images have been studied as markers for cerebrovascular events. Retinal vascular abnormalities associated with incident stroke include arteriolar narrowing and reduced microvasculature fractal dimension (FD) (a measure of vasculature “complexity”) [2]. Recent advances in optical coherence tomography angiography (OCT-A) indicate that alterations in systemic circulation are mirrored and measurable in the retinal blood flow with this technology [3,4]. An automated system able to use color fundus photos or OCT-A images to identify acute stroke events could effectively act as a proxy for brain imaging. As these imaging modalities are non-invasive, do not require the injection of contrast agents and dilation of the pupil, and have optics that can be made portable, they could significantly streamline stroke care, for example by transforming any standard ambulance into a mobile stroke unit.

Several studies have been used to established associations between stroke and retinas, especially measured with fundus imaging such as: the ARIC study [5], Rotterdam scan study [6], cardiovascular health study [7], Beaver Dam Eye study [8], Blue Mountains Eye Study [9], Mild Stroke Study [10], and UK Biobank [11]. However, these images are all acquired well after the acute stroke event, making unclear if these potential imaging biomarkers can be used in the acute stroke care setting.

Two notable exceptions are the Multi-Centre Retinal Stroke study [12] and, more recently, [13]. The former acquired fundus images no later than 7 days after the stroke event, and the latter acquired OCT-A images for subjects with a median of 11 days from the first stroke event. Ref. [12] found that the microvascular network is sparser and more tortuous in the retina of subjects with ischemic stroke; however, the data used for the study were limited to fundus images. Ref. [13] identified an association between OCT-A macular density and stroke; however, their dataset contained a significant number of subjects that did not really fall into the acute stroke window (up to 7 days according to the international Stroke Recovery and Rehabilitation Roundtable [14]). Additionally, the analysis was limited to a logistic regression model without any cross-validation or a training/test split to evaluate the internal generalizability of the predictive model and using OCT-A.

**Figure 1:**
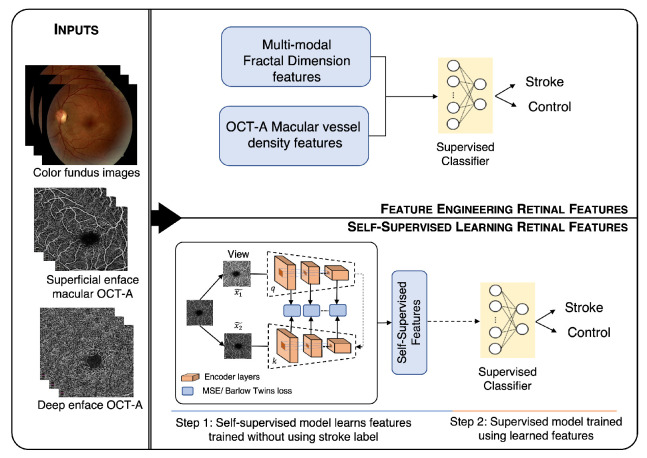
Workflow of the proposed method for stroke detection. The workflow shows two methods; the first is feature engineering (above), and the second is self-supervised learning (below). The color fundus, superficial, and deep enface OCT-A images are used as an input for both approaches. The features obtained from feature engineering and self-supervised learning are finally given to the supervised classifiers for stroke vs. control classification.

In this work, we present a study with OCT-A and fundus image acquisition in a patient population composed of stroke subjects and controls with concurrent fundus and OCT-A acquisition. We compare and contrast machine learning models trained using FD and microvasculature density as a predefined feature across modalities. Then, we use a state-of-the-art self-supervised deep learning model that creates a feature representation without any information about the stroke status of the patients, which enables us to use two additional datasets, OCT-500 [15] and ROSE [16], in addition to ours to perform a pretraining step. Then, the features learned are used to train machine learning classifiers on the stroke status of the patient. This approach allows testing of deep learning methodologies with small datasets like ours. Figure 1 shows a visual summary of our method.

## 2. Materials

The data used for this study were acquired as a part of a NASA project investigating stroke detection in deep space missions. The study was performed in accordance with the guidelines from the Declaration of Helsinki, and it was approved by the UTHealth IRB with Protocol HSC-MS-19-0352.

We included patients who were diagnosed with acute stroke between 2019 and 2021 at the Memorial Hermann Texas Medical Center, Houston, TX, USA. Houston has one of the most-diverse populations in the U.S., including a large percentage of African Americans (22.9%), Asians (6.7%), and Hispanics (44.5%) (2018 U.S. Census estimations), benefiting the generalizability of the algorithms developed. Images were acquired using the OptoVue iVue fundus camera and OptoVue Avanti OCT camera with OCT-A reconstruction software (AngioVue). In both cases, no pupil dilation or contrast agents were used, and two images per eye were acquired, one disc-centered and one macula-centered. Each OCT-A volume has a size of 640×400×400 pixels. Fundus images were acquired with a $45^{\circ }$ field of view (FOV) with a resolution of 2592×1944 pixels and were stored in jpg format. Trained graduate research assistants acquired the images after patient stabilization, clinical evaluation, and having obtained informed consent. For the purpose of these analyses, the inclusion criteria were: male or female between 18 and 99 years of age and patients arriving in the emergency department, presenting with a deficit concerning for acute stroke or controls who voluntarily participated in the study. The exclusion criteria were: physical or cognitive inability to undergo nonmydriatic retinal photography or OCT-A, inability to obtain consent from the subject’s legally authorized representative or next of kin if the subject was not able to provide assent or consent, and congenital or acquired ophthalmologic diseases that may not allow collecting gradable images, including but not limited to cataracts, retinal laser surgery, vitreous hemorrhage, retinal hemorrhage, ocular albinism, and retinitis pigmentosa. The Retina Reading Center at the University of Wisconsin-Madison masked to the presence of stroke adjudicated OCT-A images for quality and any abnormalities.

The diagnosis of ischemic stroke was based on the criteria of the Trial of Org 10172 in Acute Stroke Treatment (TOAST) classification [17] and determined by board-certified Vascular Neurologists.

### Study Population

Overall, 112 retina images were included. They were acquired from 16 patients with stroke (ischemic—15 and hemorrhagic—1), and 73 control subjects were included in the study. Demographics data for the full dataset are provided in Table 1. The younger control subjects included in the study and the presence of a hemorrhagic stroke could affect the analysis. Thus, we created another age-stroke-controlled cohort by excluding subjects younger than 45 and hemorrhagic subjects, as shown in Table 2. The time gap between the occurrence of stroke and the OCT-A examination was less than 5 days for 15 subjects and 17 days for one subject.

## 3. Methods

Figure 1 shows the overall workflow of the proposed method. The workflow shows two methodologies, (1) feature engineering (above) and (2) self-supervised learning (below). The color fundus, superficial, and deep enface OCT-A images were used as the input for both the approaches. The features obtained from the feature engineering and self-supervised learning approach were finally given to the supervised classifiers for stroke vs. control classification.

### 3.1. Feature Engineering

**Figure 2:**
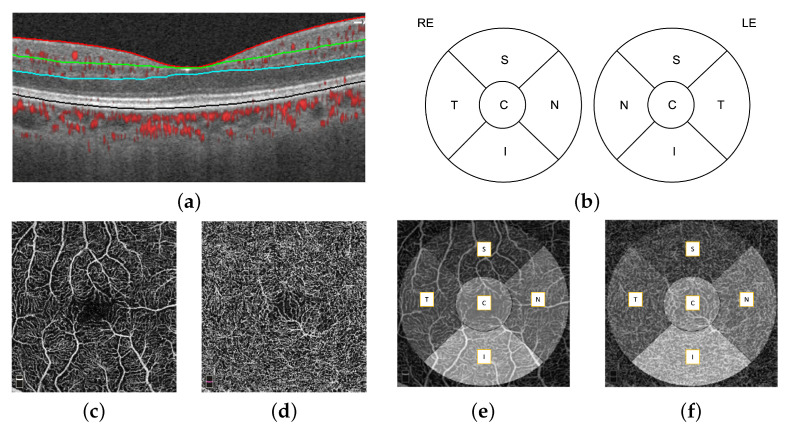
A 3×3 mm fovea-centered OCT-A image, (**a**) A horizontal OCT-A scan showing segmented retinal layers, (**b**) ETDRS circles for the right (RE) and left (LE) eye, (**c**,**e**) the en face image of the superficial layer (ILM to IPL) overlaid with the ETDRS grid, and (**d**,**f**) the en face image of the deep layer (IPL to OPL) overlaid with the ETDRS grid.

The automatic retinal layer segmentation, en face projection through selected layer ranges was performed by the OptoVue AngioVue software. First, four retinal layers were segmented, which were the internal limiting membrane (ILM), inner plexiform layer (IPL), outer plexiform layer (OPL), and retinal pigment epithelium (RPE), using the split-spectrum amplitude-decorrelation (SSADA) algorithm [18], shown in Figure 2a. The microvasculature density (defined as the proportion of perfused vasculature area over the total area measured) of the fovea-centered superficial layer and deep layer was measured automatically by the OptoVue software installed in the camera and vetted by the reading center. The superficial and deep layer did not always have the same definition. In our work, we define the superficial layer as the ILM to IPL and the deep layer as the IPL to OPL. A single microvasculature density value for each macular area (S: superior; N: nasal; I: inferior; T: temporal; C: central) was obtained. The macular areas are defined by the Early Treatment Diabetic Retinopathy Study (ETDRS) circle, as shown in Figure 2b. The 2D superficial layer (Figure 2c) and deep layer (Figure 2d) enface projections of the 3×3 mm OCT-A images overlaid with the ETDRS circle is shown in Figure 2e,f respectively.

In order to better evaluate the prediction power of the features, we used a simple ML approach, where principal component analysis (PCA) was used to reduce the feature dimensionality, then we classified each retina independently with a k-nearest neighbors (KNNs) model, and finally, we averaged the probabilities of the two OCTA enface images for each patient. The dimensionality reduction of the PCA algorithm was estimated such that at least 95% variance of the feature matrix was preserved. The KNN model was trained using k = 9, which was empirically chosen in the first experiment and, then, kept for all the subsequent ones and a leave-one-subject-out (LOSO) validation strategy. This strategy allowed us to greatly reduce the risk of data leakage between the training and test folds and to make full use of our dataset.

### 3.2. Feature Engineering with Fractal Dimension

The fractal dimension (FD) is a method of quantifying the overall complexity and density of the branching pattern of the retinal vessels. Changes in cerebral vasculature are reflected in the FD values. The dense vessels with increased branching complexity give a higher FD value, while sparse vessels with less branching complexity give a lower FD value. This retinal vasculature metric can be used as a biomarker for stroke detection. To measure the FD, first, we segmented the retinal blood vessel from fundus and OCT-A images. Vessel segmentation of fundus images was performed using the Iternet [19] algorithm. It consists of n iterations of mini-UNets after a UNet with weight-sharing and skip-connections. This consolidation outputs reliable blood vessel segmentation by finding and fixing possible defects in the intermediate results. Vessel segmentation of OCT-A images was performed using the OCTA-Net [16] algorithm. It is a two-stage framework. In the first stage, the primary segmentation results are obtained by using a split-based coarse segmentation module with ResNeSt as a backbone network. In the second stage, the segmentation results are improved by adopting a split-based refined segmentation module that utilizes original images, as well as the results from the first stage. To obtain FD values on both fundus and OCT-A images, we used three different reference fractal characterization schemes, namely the box, information, and correlation dimensions, on our segmented vasculature structure [20].

### 3.3. SHAP Analysis

Shapley additive explanations (SHAPs) [21] calculates the contribution of each feature to the prediction, which explains the machine learning model’s prediction. It does this by computing Shapley values from coalitional game theory. Shapley values are weights assigned to the model features. They show how each feature impacts the result prediction.

### 3.4. Self-Supervised Learning

Self-supervised learning helps to learn feature representations from large unlabeled datasets. This can be used as a pre-trained initialization point for different downstream tasks. We used two additional datasets, OCT-500 [15] and ROSE [16], in addition to ours, and self-supervised learning allowed us to leverage them even without having the stroke–no-stroke label. To learn the self-supervised features, we used the self-supervised method by [22]. In contrastive self-supervised learning, the idea is to keep similar samples close together, while dissimilar ones far apart. In this work, the idea of contrastive learning was extended to improve the learning of the network parameters by proposing the encoder to learn augmentation-invariant representations, not only at the end of the encoder (as done in earlier contrastive learning approaches), but also for intermediate layers. The momentum contrastive (MoCo) method [23] was used as the base self-supervised learning method. More information about the self-supervised losses used is available in Appendix B.

We used ResNet-50 [24] as the backbone architecture to learn the self-supervised features. After each of the four ResNet blocks, an intermediate feature loss was applied. This intermediate loss was added to the contrastive loss using a scaling factor. The scaling factors for the MSE and BT loss were 0.25 and $5\times 10^{-5}$, respectively. These scaling factors were selected in order to make a contrastive loss and intermediate losses in the same order of magnitude for the initial epochs. The network was pre-trained for 100 epoch using a batch size of 16. The data augmentations used for training the models were: random color jittering with the brightness, contrast, and saturation factors chosen uniformly from [0.6, 1.4] and the hue factor chosen uniformly from [−0.1, 0.1]. This augmentation was applied with 80% probability. Random Gaussian blurring with sigma was chosen with a uniform distribution between 0.1 and 2.0. Random rotation was chosen between 0 to 30 degrees. Random gray scaling was chosen with a 20% probability. Horizontally flipped images were chosen with a 50% probability. Other hyperparameters used and their respective values were: learning rate = 0.3, number of negative pairs = 65,536, embedding dimension = 128, encoder momentum = 0.99, temperature scaling = 0.07, SGD momentum = 0.9, and weight decay = $10^{-4}$.

For the downstream task (stroke vs. control classification), the features generated were used to train different supervised classifiers, which explicitly made use of the stroke labels as in the feature engineering experiments, which were compared and contrasted.

The dimensionality of the feature matrix was performed using PCA. We kept the components that explained at least 95% of the variance. This allowed the dimensionality to be manageable by all of the supervised classifiers. The classifier was tested and feature relevance was evaluated using LOSO validation. The performance measures are reported with a 95% confidence interval, which was calculated using 1000 bootstraps of the probabilities generated in the test folds.

## 4. Result

### 4.1. Macular Capillary Plexus

The comparison of the mean ± SD macular microvasculature density calculated on all the OCT-A images in stroke and control groups is shown in Table 3, where n denotes the number of subjects. The macular microvasculature density was significantly different in the superficial layers for almost all segmented areas, except the fovea (C), where no vasculature is normally expected, and any value was typically due to noise or imprecision of the segmentation algorithms. In the deep layer, the macular microvasculature density followed a similar trend, but it reached statistical significance only on Para-S, Para-N, and Para-I. The statistical significance and p-values were estimated with the two-sided Mann–Whitney U-test to reject the null hypothesis that the stroke and control populations are equal for the specific microvasculature density variable.

**Figure 3:**
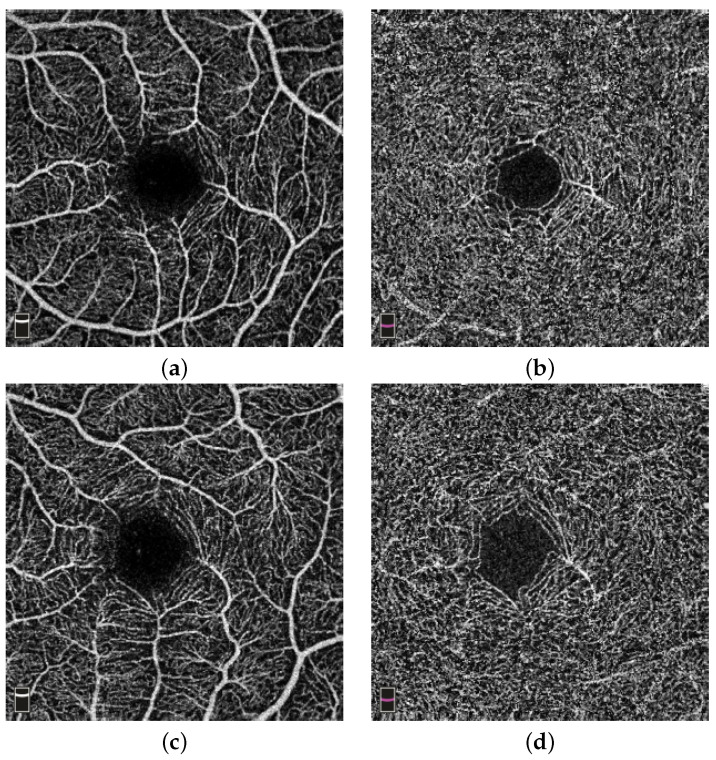
Example of microvasculature density. (**a**,**b**) Microvasculature density in the superficial (ILM-IPL) and deep layer (IPL-OPL) of the retina in a stroke subject; (**c**,**d**) vessel density in the superficial (ILM-IPL) and deep layer (IPL-OPL) of the retina in a control subject.

The higher values of the mean microvasculature density in the fovea (C) region and overall deep layer of stroke subjects were due to the use of the ETDRS circle, which is only a rough estimation of the avascular fovea zone. Figure 3a,b show an example of the microvasculature density in the superficial and deep layer of the retina in stroke subjects, respectively. The subject shown is a 28-year-old female. Figure 3c,d show examples of the microvasculature density in the superficial and deep layer of the retina in a control subject, respectively. The subject shown is 46-year-old female.

### 4.2. Feature Engineering Analysis

**Figure 4:**
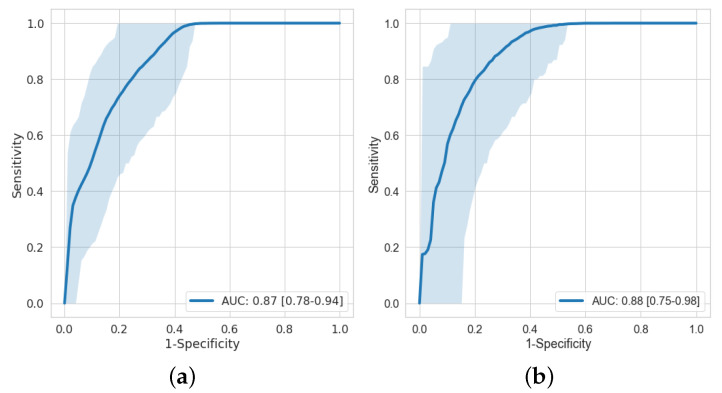
ROC curve on feature engineering with (**a**) macular microvasculature density features and (**b**) age-stroke-controlled macular microvasculature density features.

The analysis was performed on macular microvasculature density features, FD features, and their combination. Figure 4 shows the receiver operating characteristic (ROC) curves on macular microvasculature density features and age-stroke-controlled macular microvasculature density features. The AUC on macular microvasculature density features and age-stroke-controlled macular microvasculature density features with their confidence interval is 0.87 [0.78–0.94] and 0.88 [0.75–0.98], respectively. An acceptable AUC was obtained even after age matching.

Table 4 shows the AUCs with their confidence interval on different features on age-stroke-controlled and full cohorts. The columns OCT-A and Fundus represent which images were used to obtain the features. The AUC of FD features evaluated on fundus images on the full cohort were not statistically relevant as compared to the AUC of FD features evaluated on the OCT-A images. Furthermore, the AUC of FD evaluated on the fundus and OCT-A images along with macular microvasculature density did not improve the performance of the model; it was the same as that of only macular microvasculature density being used. The AUC on the age-stroke-controlled cohort with FD evaluated on both the fundus and OCT-A images was not statistically significant, and the FD features did not appear to improve the results.

In addition to accounting for age by stratifying the dataset, we performed statistical analysis using a logistic regression model with age and model probability as independent variables and stroke status as the dependent variable. This allowed us to look at the odds ratio for the model probability corrected for age. The results are in line with the previous analysis, i.e., only models including MVD features maintained an excellent predictive power when correcting for age. A detailed table with the *p*-values, confidence intervals, and odds ratios is available in Appendix A.

### 4.3. SHAP Analysis

**Figure 5:**
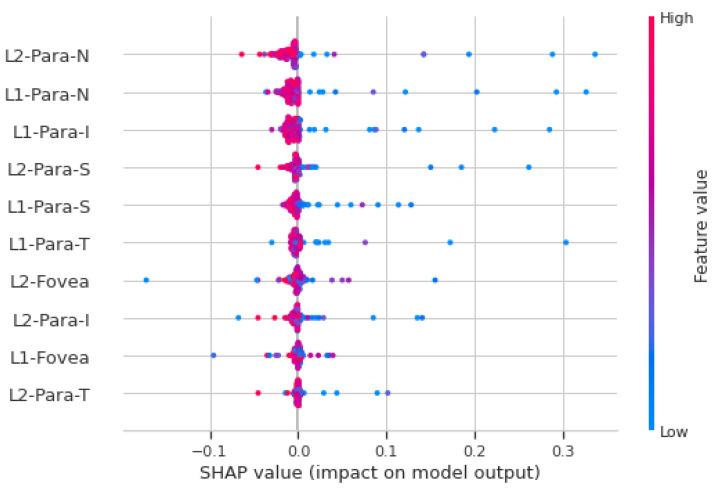
SHAP analysis summary plot for macular microvasculature density features. The color represents the value of the feature from low (red) to high (blue). The y-axis shows features ranked in descending order of importance. The x-axis shows the Shapley value and its impact on the model output.

Figure 5 shows the SHAP analysis summary plot for macular vasculature features on the full cohort. Each point on the plot is a Shapley value for a feature (variable), which are distributed horizontally. If the density of Shapley values at a particular point is high, then they are stacked vertically. The color represents the value of the feature from low to high. If the value of a feature for a particular instance is relatively high, it appears as a red dot, and relatively low feature values appear as a blue dots. Features are ranked in descending order of importance on the y-axis. The x-axis shows whether the effect of Shapley value is associated with higher/lower prediction and model impact. The features shown on the y-axis in Figure 5 are the superficial layer (L1) and deep layer (L2) with five (C, S, N, I, T) macular areas defined by the ETDRS circle. For example, L1-para-N represents the superficial layer nasal microvasculature density feature. In Figure 5, a high value of “L2-para-N” has a high and negative impact on the stroke prediction, and thus, it is negatively correlated with the target variable. The “high” comes from the red color, and the “negative” impact is shown on the x-axis. This shows that, for the vast majority of features, low vasculature density is associated with acute stroke prediction, which is consistent with the finding using the feature engineering approach.

### 4.4. Self-Supervised Learning

The self-supervised learning network was pre-trained on our dataset with two additional datasets, OCT-500 [15] and ROSE [16].

OCT-500 is a multi-modality dataset; it contains 500 subjects with 2 FOV types, including OCT and OCT-A volumes, 6 types of projections, 2 types of pixel-level label, and 4 types of text labels. The ROSE dataset contains 229 OCT-A images with vessel annotations at either the centerline level or pixel level. Both datasets do not provide any stroke or control labels. Self-supervised learning allowed us to learn the representations by training the model on data without any labels. For the downstream task (stroke vs. control classification), the representations learned by the model using the standard (MoCo + MSE and MoCo + BT) were given to different classifiers for fine-tuning.

The classifiers used for the evaluation were KNNs, decision tree, random forest, multi-layer perceptron (MLP), adaBoost, and Gaussian naive Bayes. The performance of the models on different classifiers for full and age-stroke-controlled cohorts is shown in Table 5 and Table 6, respectively. Due to the stochastic nature of the decision tree, random forest, and MLP classifiers, the AUC reported is the median of 10-fold cross-validation. From Table 5, MoCo + MSE gave better performance as compared to MoCo + BT. The best performance with AUCs of 0.81 and 0.66 was given by KNNs on the full and age-stroke-controlled cohorts, while the feature engineering approach gave an AUC of 0.87 and 0.88 on the full and age-stroke-controlled cohorts. The self-supervised learning model without using any labels for the training of the network gave comparable results with the feature engineering approach only for the full dataset, while it underperformed it for the age-stroke-controlled dataset.

## 5. Discussion

To the best of our knowledge, the presented study is the first one to evaluate the retinal microvasculature in subjects with a scan time close to the occurrence of stroke using OCT-A images, combining different modalities and evaluating feature engineering models with self-supervised approaches. OCT-A offers a unique window to non-invasively investigate the capillary network of the superficial and deep layer with high resolution and three-dimensional microvasculature imaging of the retina, which is absurd in fundus images, providing only a two-dimensional view [25]. Thus, the OCT-A images can provide more accurate information about retinal microvascular abnormalities. These images are captured without any radiation exposure (which is required in CT) and are cost-effective. Automatic segmentation of retinal layers, regions, and microvasculature density calculation was performed using the built-in software in the camera, which allowed direct observation and reduced measurement errors.

Previous studies had reported individual associations between stroke and specific parameters. The most-studied parameters in the retina are enhanced arteriolar light reflex, narrower arteriolar caliber, wider venular caliber, increased arteriolar and venular tortuosity, arteriovenous nicking, decreased retinal FD, decreased central retinal arteriolar equivalent, increased central retinal venular equivalent, and decreased arteriole-to-venule ratio, and the presence of localized RNFL defects are associated with ischemic stroke, compared with controls [12,26,27,28,29]. However, vessel calibers’ measurements from a single time point may not be effective for stroke analysis because of pulse variation. FD and microvasculature density measurements are attractive because they reflect blood distribution optimality and are relatively static. Recently, Reference [13] proposed an approach that detects the changes in retinal microvasculature in patients with stroke. However, the images were acquired 1–419 days after the stroke attack, limiting their use for acute stroke detection.

In this study, the retinal microvasculature in subjects with stroke and healthy controls was quantitatively measured on OCT-A images. We demonstrated that the quantitatively measured microvasculature density in different layers and macular regions of the OCT-A is different in subjects with stroke compared with healthy control patients. We observed that the microvasculature density in macular regions was significantly reduced in subjects with stroke. Feature engineering was performed using these microvasculature densities and FD features for stroke vs. control classification. Furthermore, we also demonstrated the performance of the self-supervised learning technique for stroke vs. control classification.

The macular capillary plexus analysis revealed a decrease in the retinal microvasculature density in subjects with stroke compared to healthy controls; however, for stroke identification, we cannot only rely on this information as it is dependent on accurate detection of the ETDRS circle, which is only a rough estimation of the avascular fovea zone. Further, the feature engineering analysis showed that the integration of all macular microvasculature density features derived from OCT-A with machine learning models could act as an effective biomarker of stroke identification. Given that young control subjects were included in the study, we could not rule out the possibility of this affecting the analysis. Thus, in a further analysis, we showed that, even after using the features from the age-stroke-controlled subjects, the performance was not affected, with an AUC of 0.88. The retinal FD is static and has been used as a biomarker for the detection of diseases such as diabetes and hypertension. We performed the analysis by integrating macular microvasculature density features from OCT-A images and FD features from fundus and OCT-A images. The FD features did not provide any additional information about microvascular changes beyond the microvasculature density features. Furthermore, the SHAP analyses found that subjects with a lower parafovea microvasculature density of the deep layer were less likely to be classified as stroke. Finally, the ROC analysis of self-supervised learning showed that the KNN classifier evaluated on features extracted from the self-supervised learning (MoCo + MSE) technique gave AUCs of 0.81 and 0.66 on the full and age-stroke-controlled cohorts. In the medical imaging domain, a small labeled data setting is common, and this is because manual annotation of the images is a time-consuming and expensive process. Thus, the performance achieved by our approach becomes more important. However, the drop in performance of the self-supervised method between the two datasets can be indicative of the model learning confounders rather than the effect of the retina induced by acute stroke. This hypothesis is further corroborated by the fact that the feature engineering approach on macular microvasculature density features derived from OCT-A did not suffer from any drop in performance. Overall, the results of all the analyses showed with greater certainty the usefulness of the macular microvasculature density features as potential biomarkers for acute stroke, which can act as a proxy to avoid brain imaging.

Our work has some limitations. First, while only high-quality OCT-A images were included in the dataset, corresponding fundus images with small artifacts and not of high quality were included, in order to avoid further reducing the size of the dataset. Additionally, we only employed macula-centered fundus images and not optic-disc-centered fundus images. More importantly, while encouraging results were obtained, further work is needed to be able to demonstrate that even the best-performing methods can be used as a diagnostic system, as we have not demonstrated that we can distinguish ischemic from hemorrhagic stroke, which will be essential for using this as system for the prompt delivery of the appropriate medications, such as tissue plasminogen activator (or tPA). Similarly, our dataset is too small to evaluate the different effects on the retina vasculature of different types of ischemic events and stroke locations. Finally, for some subjects, we were able to acquire only a single retina, as standard OCT-A/fundus cameras struggle with subjects that cannot keep their eyelid open, which is a common sign of stroke. This did not allow us to evaluate a correlation between macular vessel density and other features ipsilateral to the hemisphere affected by stroke. This limitation of the current commercial cameras could be solved by a design that couples the camera with a mechanical system able to keep the eyelid open.

## 6. Conclusions

The study compared and contrasted multiple methodologies, on multi-modal retina imaging, that could be used for acute stroke diagnosis using OCT-A and fundus images. The performance of feature engineering and self-supervised learning retinal features using OCT-A and fundus images was shown. The study indicated that the decreased macular microvasculature density, signifying a sparser vessel network, was associated with acute stroke in this cohort. These findings suggest that the macular microvasculature density features could detect changes due to acute stroke; however, further work is needed for the final proof for a diagnostic system.

## Figures and Tables

**Table 1:** Demographics for full cohort.

Stroke	Age	Sex		Race				Total Subjects
		Male	Female	White	Black	Asian	Other	
control	39.63 ± 14.13	31	42	34	16	20	3	73
stroke	58.25 ± 14.47	8	8	13	2	1	0	16

**Table 2:** Demographics for age-stroke-controlled cohort. Only ischemic stroke subjects are included.

Stroke	Age	Sex		Race				Total Subjects
		Male	Female	White	Black	Asian	Other	
control	57.81 ± 11.73	7	14	4	11	4	2	21
stroke	58.13 ± 14.94	7	8	13	2	0	0	15

**Table 3:** Comparison of mean ± SD microvasculature density in stroke and control groups. n is the number of subjects in that group (stroke or control) on which the mean vasculature density was calculated. Statistical significance of the difference between variables computed with the Mann–Whitney U-test. n.s: not significant, *: *p* < 0.05, **: *p* < 0.01, ***: *p* < 0.001.

Microvasculature Density	Superficial Layer			Deep Layer		
	Stroke *n* = 16	Control *n* = 73		Stroke *n* = 16	Control *n* = 73	
Fovea (C)	18.12 ± 8.14	17.49 ± 5.60	n.s.	40.09 ± 17.04	33.63 ± 6.67	n.s.
Para-T (T)	44.52 ± 7.16	49.95 ± 2.71	**	56.54 ± 12.40	57.06 ± 2.71	n.s.
Para-S (S)	45.33 ± 12.66	52.88 ± 3.01	***	55.90 ± 12.59	57.64 ± 2.66	*
Para-N (N)	43.41 ± 12.50	50.43 ± 3.92	**	55.75 ± 13.20	57.43 ± 3.02	*
Para-I (I)	45.48 ± 13.12	52.71 ± 3.05	**	54.91 ± 13.27	57.04 ± 3.36	*
Unsegmented	196.86 ± 53.59	223.47 ± 18.28	n.s	263.20 ± 68.50	262.79 ± 18.41	n.s.

**Table 4:** AUCs with their confidence intervals on different features and their combinations with full and age-stroke-controlled cohorts. The columns OCT-A and Fundus represent which images were used to obtain the features.

Cohort	Features	OCT-A	Fundus	AUC [Confidence Interval]
Full	MVD	Yes	No	0.87 [0.78–0.94]
	FD	No	Yes	0.57 [0.21–0.65]
	FD	Yes	No	0.70 [0.52–0.88]
	FD	Yes	Yes	0.68 [0.51–0.86]
	MVD + FD	Yes	Yes	0.87 [0.78–0.94]
	MVD + FD	Yes	No	0.87 [0.78–0.94]
	MVD + FD	Yes	Yes	0.87 [0.78–0.94]
Age-Stroke-Controlled	MVD	Yes	No	0.88 [0.75–0.98]
	FD	No	Yes	0.54 [0.27–0.68]
	FD	Yes	No	0.60 [0.37–0.82]
	FD	Yes	Yes	0.72 [0.54–0.90]
	MVD + FD	Yes	Yes	0.87 [0.75–0.98]
	MVD + FD	Yes	No	0.88 [0.75–0.98]
	MVD + FD	Yes	Yes	0.87 [0.75–0.98]

MVD: macular microvasculature density.

**Table 5:** Performance of models trained via self-supervised learning methods (MoCo + MSE and MoCo + BT) for the stroke vs. control classification task after fine-tuning on different classifiers. Full cohort is used.

	AUC	
**Classifier**	**MoCo + MSE**	**MoCo + Barlow Twins**
KNNs	0.81 [0.68–0.92]	0.70 [0.55–0.82]
Decision Tree	0.62 [0.46–0.78]	0.55 [0.40–0.68]
Random Forest	0.74 [0.59–0.87]	0.71 [0.57–0.85]
MLP Classifier	0.61 [0.45–0.76]	0.71 [0.55–0.86]
AdaBoost	0.62 [0.44–0.80]	0.71 [0.54–0.86]
Gaussian Naive Bayes	0.76 [0.63–0.89]	0.68 [0.50–0.84]

**Table 6:** Performance of models trained via self-supervised learning methods (MoCo + MSE and MoCo + BT) for stroke vs. control classification task after fine-tuning on different classifiers. Age-stroke-controlled cohort is used.

	AUC	
**Classifier**	**MoCo + MSE**	**MoCo + Barlow Twins**
KNNs	0.66 [0.46–0.85]	0.61 [0.41–0.80]
Decision Tree	0.60 [0.42–0.78]	0.59 [0.22–0.60]
Random Forest	0.57 [0.38–0.77]	0.51 [0.31–0.69]
MLP Classifier	0.63 [0.57–0.81]	0.41 [0.22–0.64]
AdaBoost	0.54 [0.24–0.70]	0.42 [0.23–0.61]
Gaussian Naive Bayes	0.52 [0.31–0.77]	0.53 [0.32–0.75]

## Data Availability

The trained model is available at https://glabapps.uth.edu/ (accessed on 7 December 2022).

## Abbreviations

The following abbreviations are used in this manuscript:

BT	Barlow twins
CT	computed tomography
ETDRS	Early Treatment Diabetic Retinopathy Study
FD	fractal dimension
FOV	field of view
I	inferior
ILM	internal limiting membrane
IPL	inner plexiform layer
KNNs	k-nearest neighbors
LOSO	leave-one-subject-out
MLP	multi-layer perceptron
MoCo	momentum contrastive
MRI	magnetic resonance imaging
MSE	mean-squared error
N	nasal
OCT-A	optical coherence tomography angiography
OPL	outer plexiform layer
PCA	principal component analysis
RPE	retinal pigment epithelium
S	superior
SHAP	Shapley additive explanations
SSADA	split-spectrum amplitude-decorrelation
T	temporal
TOAST	Trial of Org 10172 in Acute Stroke Treatment

## Appendix A. Feature Engineering Analysis

**Table A1:** *p*-values and odds ratios of different features and their combinations on age controlled subjects. The columns OCT-A and Fundus represent which images were used to obtain the features.

Features	OCT-A	Fundus	*p*-Value	Confidence Interval (2.5–9.75)%	Odds Ratio
MVD	Yes	No	0.016	2.735–18,151.842	222.806
FD	No	Yes	0.293	0.001–7.654	0.095
FD	Yes	No	0.012	4.039–98,411.343	630.440
FD	Yes	Yes	0.112	0.485–1060.619	22.669
MVD + FD	Yes	Yes	0.014	3.228–30,092.671	311.659
MVD + FD	Yes	No	0.016	2.735–18,151.842	222.806
MVD + FD	Yes	Yes	0.014	3.228–30,092.671	311.659

MVD: Macular microVasculature Density.

## Appendix B. Self-Supervised Losses

The feature representation learned by the standard MoCo is achieved by minimizing the InfoNCE loss, given as follows:

(A1) $$L_{InfoNCE}\left(x\right)=-log\frac{exp(q\left(\tilde{x_{1}}\right)\cdot k\left(\tilde{x_{2}}\right)/\tau )}{exp\left(q\left(\tilde{x_{1}}\right)\cdot k\left(\tilde{x_{2}}\right)/\tau \right)+\sum_{i=0}^{N}exp\left(q\left(\tilde{x_{1}}\right)\cdot k\left(\tilde{z_{i}}\right)\right)/\tau }$$

where $\tilde{x_{1}}$ and $\tilde{x_{2}}$ are two different views (called positive pair), which are generated by the augmentation of the same image x. There are N negative pairs of $\tilde{x_{1}}$ and $\tilde{z_{i}}$, where $\tilde{z_{i}}$ is the random augmentation of a different image. The parameters of the encoder *q* are optimized by minimizing $L_{InfoNCE}$. Encoder *k* is an identical copy of encoder *q*, whose parameters are an exponential moving average of *q*. τ is a temperature-scaling hyper-parameter.

In addition to contrastive loss, two additional loss terms were used. The first loss function used is based on the mean-squared error (MSE), and the second is the Barlow twins (BT) loss [30]. These additional loss functions help the network in learning augmentation-invariant features early in the model and, thus, give high-quality features. The MSE was computed on intermediate features between two views of the same image. These extracted intermediate features are passed through an adaptive average pooling layer. This was performed to reduce the spatial variance in the positive pair that might be caused due to augmentation. BT loss is designed to bring the distance between the cross-correlation matrix of two feature representations and the identity matrix close. The BT loss function is defined as follows:

(A2) $$\begin{array}{cc} L_{BT}=\underset{\mathrm{invariance}\;\mathrm{term}}{\underset{︸}{\sum_{i}{\left(1-C_{ii}\right)}^{2}}}+\lambda \underset{\mathrm{redundancy}\;\mathrm{reduction}\;\mathrm{term}}{\underset{︸}{\sum_{i}\sum_{j\neq i}C_{ij}{}^{2}}} & C_{ij}=\frac{\sum_{b}z_{b,i}^{A}z_{b,j}^{B}}{\sqrt{\sum_{b}{\left(z_{b,i}^{A}\right)}^{2}}\sqrt{\sum_{b}{\left(z_{b,j}^{A}\right)}^{2}}} \end{array}$$

where λ is a weight that trades off between the first and second terms of the loss function. Each $C\in {\left[-1,1\right]}_{}^{d\times d}$ is the cross-correlation matrix computed between the outputs of the two identical networks of batch dimension *d*. Here, $z^{A}$ and $z^{B}$ are a batch of intermediate feature representations for two augmented versions of the same image with batch size *b*. *i* and *j* are the vector dimensions of the network’s output. The objective of the invariance term is to try to equate the diagonal elements of the correlation matrix to one, which in turn, makes the representation invariant to augmentation. The objective of the redundancy term is to try to equate the non-diagonal elements of the correlation matrix to zero, thereby decorrelating the different components of the feature representation. This decorrelation helps to reduce the redundancy between output units and, thus, it contains non-redundant information. The intermediate feature representations are passed through an adaptive average pooling layer to obtain a feature vector. This feature vector is passed through three linear layers with the first two layers, followed by ReLU non-linearity and batch normalization. The resultant output representations are given to the $L_{BT}$ function.
